# The Relationship between a Rotational Molding Processing Procedure and the Structure and Properties of Biobased Polyethylene Composites Filled with Expanded Vermiculite

**DOI:** 10.3390/ma15175903

**Published:** 2022-08-26

**Authors:** Joanna Aniśko, Mateusz Barczewski, Adam Piasecki, Katarzyna Skórczewska, Joanna Szulc, Marek Szostak

**Affiliations:** 1 Institute of Materials Technology, Faculty of Mechanical Engineering, Poznan University of Technology, Piotrowo 3, 61-139 Poznan, Poland; 2 Institute of Materials Engineering, Faculty of Materials Engineering and Technical Physics, Poznan University of Technology, Piotrowo 3, 61-138 Poznan, Poland; 3 Faculty of Chemical Technology and Engineering, Bydgoszcz University of Technology, Seminaryjna 3, 85-326 Bydgoszcz, Poland

**Keywords:** rotational molding, bio-polyethylene, composite, vermiculite

## Abstract

Rotational molding is a technology in which polymeric thin-walled products can be made. The newest descriptions of this technology concern the possibility of obtaining polymer composite materials. There are two main methods of incorporating fillers into a polymer matrix. Dry blending is based on mixing fillers with polymer powders before rotational molding by hand or using automatic mixers. In the melt compounding method, fillers are mixed with the polymer in the preliminary step by melt processing and then grinding or pulverization to obtain polymer powders for rotational molding. This work aimed to investigate the impact of the processing procedure on the structure and properties of biobased composites with expanded vermiculite. Produced rotomolded parts were examined using mechanical tests to assess changes in tensile, flexural, and impact properties. The most significant difference in mechanical properties was noted for samples with 10 wt% expanded vermiculite (EV). The elasticity modulus increases by almost 2 fold when the sample is prepared in a two-step process, the tensile strength is 4-fold higher, the flexural modulus is 3-fold higher, and the flexural strength is 5-fold higher. We also investigated thermomechanical properties in DMA measurement. The void volume content was also measured to control the quality of obtained parts. The porosity of dry blended samples containing more than 2 wt% EV is almost 2-fold higher. Other methods to control quality and structure were optical and scanning electron microscopy used for rotomolded parts and polymer powders. The investigations of rotomolded parts were supplemented with a complete description of used materials, including the particle size distributions of polymer powders and filler. Analysis of the thermal properties and chemical structure was also performed despite all the mechanical tests. The emerging conclusions from the research clearly show that the two-step process allows for achieving a more beneficial mechanical performance of the composites made of the biobased polymer in rotational molding technology.

## 1. Introduction

Rotational molding technology is used to obtain thin-walled products from thermoplastic polymeric powder. Due to its relatively simple processing procedure and low-cost tools, this technology has largely displaced the technologies of blown extrusion, thermoplastic polymers, and thermoset composites that have been used so far. The polymer powder is placed in a heated mold reflecting the external surface of the part and subject to rotational movement along the horizontal and vertical axes. The process occurs in four steps: loading the polymer powder, heating the mold, cooling, and demolding the finished product [1]. All stages in this technology occur in the same mold via a heating mold in the heating chamber above the polymer melting temperature and subsequent cooling in the cooling station. This simplicity of the process can be a drawback because of the complex control of the process parameters inside the mold [1]. To obtain the rotomolded parts effectively, a few process conditions have to be considered in this technology. The molds for rotational molding should have venting to prevent the high pressure from distorting the rotomolded parts. The inner surface of the molds should be coated with release agents to help demolding. The speeds of the rotating axes must be carefully selected to produce the desired rotomolded part [1]. Because of the character of this procedure, products formed in this technology are characterized by almost no internal stresses [2,3]. This process is a low-pressure production method, and low-shear rates occur while processing. Other advantages are the possibility of producing large hollow objects without joints and less material is wasted compared to different plastic processing technologies. The most significant disadvantage is the high material cost because of the complicated grinding of polymer materials [1,4,5]. Much attention is paid to carefully prepared polymer powder for this technology because the particle powder size is a crucial characteristic of materials in this technology. According to Crawford [2], the most processable polymer powder should have a narrow particle size distribution and a size below 500 μm. Additionally, it is ideal if the particle size distribution curve is Gaussian like. These properties are difficult to obtain because polymer materials are fragile to elevated temperatures, which can occur while grinding [6].

When preparing composites in rotational molding technology, there are two different methods of incorporating the reinforcement: single-step dry blending and preliminary melt processing [7]. Dry blending is a single-step process based on mechanical mixing of the fillers with a polymer powder matrix, usually using automatic mixers. The dry blending process is a one-step process to obtain composite materials ready to be placed in forms for rotational molding. So far, published studies about rotomolded composites prepared by dry blending usually discuss using the virgin polymer in a powder state, obtained from producers ready to be placed in forms for rotational molding [8,9,10]. There are examples in the literature where dry blending is used, especially for fiber-reinforced composites [10,11,12,13]. Unfortunately, due to the presence of rigid infusible filler particles, dry blended composites are often characterized by high porosity; the higher the filler content the bigger the holes. Cisneros-López et al. reported that polylactide (PLA)-agave fiber composites have high porosity, even up to 60–70% when 40 wt% of fiber filler was incorporated [14]. Another example of a dry blended rotomolded composite with agave fiber was discussed by González-López [15]. PLA composites modified with 40 wt% maleated PLA (MAPLA) agave fibers with lengths of 297–400 μm and approximately 60% porosity. Hejna et al. [3] also discuss the influence of porosity in rotomolded polyethylene composites with wheat bran, made by one-step dry blend processing. The maximum porosity is 10.86% for samples containing 20 wt% natural particle-shaped filler.

The melt compounding method requires an additional processing step, most often extrusion, to obtain composite extrudate, which becomes ground or micropelletized. Melt compounding of composites for rotational technology was used for producing composites containing graphite [16], fumed silica [17], and wollastonite [18]. The dry blending process in the preparation of fiber composites is usually obtained by an industrial blender [12], a shear mixer [19], or a knife mill [20]. Melt compounding of composite material for rotational molding is beneficial when preparing rotomolded composites with uniform filler distribution. In [17], results showed that low-density polyethylene (LDPE)-fumed silica samples manufactured from melt compounded composite powders have a good distribution of filler particles in polymer bulk after processing. The scanning electron microscopic (SEM) images confirm this uniform filler distribution [17]. It can be concluded that when the natural materials, especially fiber materials, are incorporated into rotomolded composites, dry blending preparation is often chosen [14,15]. This is because the exposition of the organic fillers on the degradative thermal environment during processing needs to be reduced. When using thermally stable inorganic fillers, the melt compounding method is reasonable. Other characteristics of fillers that distinguish the composite preparation method are particle size, distribution and aspect ratio. Published literature show that fiber-shaped materials with a length from approximately 100 to 500 µm are dry blended with a matrix before rotational molding. For example, those fillers are agave fibers of sizes 297–400 µm and 149–210 µm, and they can be successfully dry blended with polyethylene [11]. Maple fibers are used as reinforcements in the following size ranges: 125–250, 250–355 and 355–500 µm, also dry blended with a polymeric matrix [19]. On the other hand, melt compounding of fillers with the matrix is usually used to prepare composite materials with particles below the average size of approximately 100 µm. The flake graphite used to prepare a melt-blended composite has an average size of 112 µm [16]; however, another study discussed manufacturing the rotomolded composites containing smaller wollastonite particles with an average size of 16.6 µm [21]. The smallest particles used to prepare rotationally moldable composite materials are reported as from 5 to 50 nm, and this filler is fumed silica [17]. There are few exceptions from these dependences. There are studies where the inorganic and nanosized particles are dry blended with a polymeric matrix. One such example is work by Daryadel and coworkers [8], where nanoclay Cloisite 30B (size of used nanofiller is 1–2 nm) is dry blended with linear low-density polyethylene (LLDPE). In this case, there are also some remaining pores and voids in structure samples prepared at the lowest temperature.

This work aims to compare two different methods of preparing rotomolded composites. The inorganic filler (expanded vermiculite EV) was used as a reinforcement in biobased polyethylene matrix composite manufacturing. Vermiculite is a natural phyllosilicate clay, which has a layered structure of an octahedral sheet and two tetrahedral sheets, which, when heated, expand approximately 30 fold above 300 °C. It is lightweight, inexpensive, non-toxic, chemically inert, and resistant to thermal decomposition, making this material ideal as an insulator or filler in insulation applications [22,23]. When vermiculites are heated quickly, the interlayer water evaporates. This affects the formation of exfoliated flakes with the form of concertina-shaped granules. The expansion of vermiculite increases the volume by 8–12 fold [24], obtaining filler with a high aspect ratio [25]. The formation of vermiculite is based on weathering or hydrothermal alteration of hydrobiotite or phlogopite mineral phases [23]. The general chemical structure of vermiculites is X_4_(Y_2–3_)O_10_(OH)_2_M·nH_2_O, where X is a Si or Al, Y is Mg^2+^, Fe^2+^ or Fe^3+^, Al^3+^ and M is an exchangeable by Mg^2+^, Ca^2+^, Ba^2+^, Na^+^, and K^+^. X responds to a tetrahedral sheet, which is usually silica, and Y responds to an octahedral, usually magnesia [26,27].

There are reports on polyethylene–vermiculite composites based on bio-high-density polyethylene in the literature. These materials have been tested to investigate permeability, which does not deteriorate significantly after adding vermiculite [26]. The bio-polyethylene with vermiculite can be formed into a flat film in which the increase in hydrophilicity is observed with the increasing amount of vermiculite. In contrast, pure HDPE films have a hydrophobic surface [28]. Polyethylene is a non-polar polymer, but the vermiculite particles are polar. This means that there are limited interactions between these two phases. In most cases, filler treatment needs to be performed to improve the bonding of vermiculite in the PE matrix [26]. The vermiculite treated with an ionic surfactant becomes the organophilic filler, which helps obtain an even distribution of this filler in PE and modify its fire behavior [29]. It should be underlined that surface modification of the filler results in contrary modification results, the addition of unmodified vermiculite decreases the burning rate of HDPE composites, while composites made with modified fillers showed the opposite effect. The vermiculite can also be treated by a substance that has antifungal properties. This treatment provides good dispersion and adhesion to LDPE and long-lasting antifungal efficacy [30]. The PE–vermiculite composites were also investigated due to their antibacterial properties. It was confirmed that the addition of vermiculite prevents the survival and multiplication of bacterial strain Enterococus faecalis on the composite surface [31].

As part of this work, two methods of preparing composites in the rotational molding technology were conducted. The filler may be introduced by both melt mixing and dry blending. A dry blending process is used to obtain rotomolded composites filled with organic fillers, especially natural fibers. The two-step process is considered in the preparation of composites with inorganic, nanometric fillers. In this case, the mixing methods were compared for the production of composites with a complex-shaped filler. The previously mentioned nature of expanded vermiculite allows obtaining exfoliated flakes. The emerging conclusion from the literature analysis is that melt compounded composites have better mechanical properties and lower porosity. The investigations described in this work were undertaken to examine this statement thoroughly.

## 2. Materials and Methods

### 2.1. Materials

The matrix used was the biobased high-density polyethylene SHC 7260 I’m Green^®^ provided by Braskem (Sao Paulo, Brazil). It is characterized by a melt flow index (MFI) of 7.2 g/10 min (190 °C/2.16 kg) and a density of 0.959 g/cm^3^. The minimum biobased content of this polyethylene is 94%, according to ASTM D6866. The introduced filler is expanded vermiculite (EV) obtained from Perlit Polska (Puńców, Poland). The measured aspect ratio of the filler was 1.69, the BET surface area (SBET) is 12.58 m^2^/g, and the t-Plot external surface area (SEXT) is 12.77 m^2^/g. The broader characteristics of the filler, including its density and particle size distribution, have been presented in further parts of the manuscript.

### 2.2. Preparation of Samples

Before compounding, the filler was milled on a Retsch ZM 200 (Haan, Germany) grinder with a rotor speed of 6000 rpm and a 250 μm sieve. The sieve analyzer (Fritsch ANALYSETTE 3 PRO (Weimar, Germany)) distinguished the desired fraction. The particle size was less than 200 μm. The expanded vermiculite was introduced at amounts of 0.5, 1, 2, 5, and 10 wt%, which is approximately a volume content of 0.2, 0.4, 0.8, 2.2 and 4.6 %. The composites in rotational molding were prepared in two ways:

(1) Dry blended

The biobased HDPE was milled on a Retsch ZM 200 grinder to obtain the powder suitable for rotational molding. The grinding parameters were 10,000 rpm for rotary knives’ speed, and the size of the sieves was 500 μm. The introduced filler was manually blended with HDPE powder. Before the rotational molding process, materials were placed in a vacuum laboratory cabinet dryer for 12 h at 80 °C.

(2) Melt compounded

To obtain the composite materials, the extrusion process was performed using the twin-screw co-rotating extruder Zamak 16/40 EHD (Zamak Mercator, Skawina, Poland). The polyethylene, before extrusion, was milled with a high-speed grinder TRIA 25-16/TC-SL (Milan, Italy) to obtain the finest particles to improve the physical mixing process. The HDPE particles and expanded vermiculite were dried before extrusion in a laboratory cabinet dryer at 80 °C for 12 h. The extrusion process was carried out at a highest temperature of 160 °C, and the rotational speed of the screws was 150 rpm. The extrudates were cooled in forced airflow and, after this, pelletized. The obtained material was subjected to further grinding to obtain a polymer powder suitable for rotational molding technology. The grinding was performed as previously on the Retsch ZM 200 grinder. The speed of the rotor was 10,000 rpm, and the size of the sieves was 500 μm. The milled composite materials were dried at 60 °C for 12 h and then subjected to rotational molding.

### 2.3. Rotational Molding Processing

**Figure 1:**
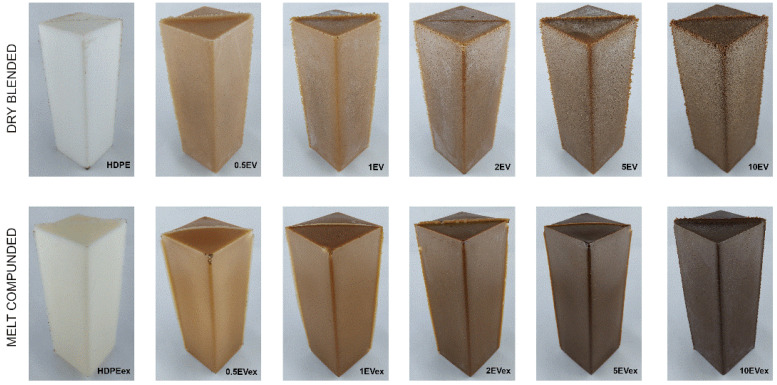
Rotomolded composites with expanded vermiculite.

The dried materials, prepared in two methods, were subjected to the rotational molding process by placing the material in the form of 2 mm thick steel sheets with dimensions of 180 × 60 × 60 mm^3^. The rotational molding machine was the laboratory scale machine: a single-arm shuttle rotational molding machine (REMO GRAF, Poznan, Poland) [32]. The rotational speed for the horizontal and vertical axes was 8 and 5 rpm, respectively. The temperature of the heating chamber was 250 °C and the time the material spent in this chamber was 20 min. The next step of the rotational molding process was the cooling process conducted over 10 min in the forced airflow. All samples were prepared with the same parameters, and the amount of material used was 100 g. The photographs of manufactured rotomolded parts are collectively presented in Figure 1. The acronyms of sample series refer to the weight content of the filler, while samples made from melt compounded polymer composite powders additionally contained in the suffix “ex”, e.g., 0.5EVex.

### 2.4. Thermal Properties and Chemical Structure

The rotomolded composites were analyzed to investigate any thermal and chemical changes. Differential scanning calorimetry (DSC), thermogravimetry (TGA), and Fourier-transform infrared spectroscopy (FTIR) were performed. The DSC measurements were carried out in the temperature range −30 to 200 °C with a heating and cooling rate of 10 °C/min and under a 20 mL/min nitrogen flow. The apparatus used for this test was Netzsch DSC 204 F1 Phoenix (Selb, Germany). The 5 mg ± 0.25 mg samples were placed in aluminum pierced pans. The thermogravimetry test was performed on a Netzsch TG 209 F1 Libra instrument (Selb, Germany). The 10 mg ± 0.5 mg samples were placed in Al_2_O_3_ crucibles and subjected to heating at a rate of 10 °C/min from 20 to 900 °C under a nitrogen flow of 20 mL/min. The last test was FTIR in the ATR mode using the Jasco FT/IR-4600 (Tokyo, Japan). The scanning resolution was 4 cm^−1^, the wavenumber range was 4000–400 cm^−1^, and the number of scans was 32.

### 2.5. Particle Size Distribution

To determine the particle size distribution of powders used in a rotational molding process and filler. All samples were analyzed using a laser particle sizer Fritsch ANALYSETTE 22 (Weimar, Germany) apparatus operated in the range of 0.08–2000 μm. The cumulative size distribution Q3(x) and adequate histogram dQ3(x) were considered during the analysis.

### 2.6. Structural Analysis

The samples’ outer and inner surfaces were analyzed using the optical microscope Levenhuk DTX 500 LCD Digital Microscope (Warsaw, Poland).

Scanning electron microscopy (SEM) was performed using the Tescan MIRA 3 (Brno-Kohoutovice, Czech Republic) microscope. Structure analysis was performed for composite powder samples and fractured rotomolded samples to investigate the distribution of expanded vermiculite in magnification of 100× and 200×, respectively. The micrographs of the filler were also examined at a magnification of 5000×. The measurements were conducted with an accelerated voltage of 12 kV in backscattered electrons (BSE) and the secondary electron (SE) mode. The thin carbon coating (~20 nm) was deposited on samples using the Jeol JEE 4B vacuum evaporator (Tokyo, Japan).

### 2.7. Density and Void Volume

The density measurement was performed using an Anton Paar Ultrapyc 5000 gas pycnometer (Graz, Austria). The filler and powders for rotational molding density were evaluated using this method. The theoretical density was calculated for samples dry blended using the Equations (1) and (2):

ρ_t_ = V_f_ · ρ_EV_ + (100 − V_f_) · ρ_HDPE_ (1)

V_f_ = (W_f_ · ρ_HDPE_)/((100 − W_f_) · ρ_EV_) (2)

where ρ_EV_ is the density of expanded vermiculite [g/cm^3^], ρ_HDPE_ is the density of matrix (high-density polyethylene) [g/cm^3^], Vf is the volume fraction of the filler [%], and Wf is the weight fraction of the filler [%].

The density of HDPE and expanded vermiculite was taken from the powders’ density measurement. The apparent and actual densities were measured using the hydrostatic method with ethanol (ρ_ethanol_ = 0.789 g/cm^3^). The density was calculated using Equation (3), where m_1_ is the mass of the sample in air and m_2_ is the mass of the sample in a fluid:

(3) $$\rho_{a}=\frac{m_{1}}{m_{1}-m_{2}}\cdot \rho_{\mathrm{ethanol}}$$

All masses were measured using the AXIS AD200 balance (Gdańsk, Poland). The density was measured for minimum 3 samples, and the average value was considered for further analysis.

To measure the porosity of samples, the densities mentioned above were used. To evaluate the value of the void volume content (P), the density of rotomolded composites (apparent density ρ_a_) was compared to the density of materials powders (actual density ρ_0_):

(4) $$P=\frac{\rho_{0}-\rho_{a}}{\rho_{0}}\cdot 100\%$$

### 2.8. The Melt Flow Index

The melt flow index (MFI) of materials obtained in the extrusion process was measured using the plastometer Dynisco D4002DE (Franklin, MA, USA). The test was carried out at a temperature of 190 °C and a load of 2.16 kg according to the ISO 1133 standard.

### 2.9. Mechanical Test

A tensile test was performed according to the standard PN-EN ISO 527 using the universal testing machine Zwick Roell Z010 (Ulm, Germany). The samples’ dimensions were 10 mm in width, 100 mm in length, and different thicknesses for each specimen. Measurements were performed with the crosshead speed of 1 mm/min during Young modulus determination (up to elongation of 0.2%) and 5 mm/min in the further part of the experiment. The reported values of tensile properties are the average of at least 5 samples. The material’s toughness was also calculated from the stress–strain curve obtained from the tensile test. Toughness is the area under the stress–strain curve [33]. All specimens used for the determination of mechanical and thermomechanical properties were conditioned before testing at 23 °C and relative humidity of 55% for 24 h.

The three-point flexural test was carried out on a universal testing machine Zwick Roell Z010 (Ulm, Germany). The measurement was evaluated according to the PN-EN ISO 178. Five samples with dimensions 10 × 80 mm^2^ and different thicknesses for each series were subjected to test to calculate the average value. The crosshead speed during flexural modulus determination was 1 mm/min and 5 mm/min during the rest of the measurements.

The Charpy impact test was performed using the Zwick/Roel Hit 25 (Ulm, Germany) testing machine. The samples were 80 mm in length and 10 mm in width with a notch. At least five samples were measured to calculate the average value of the impact strength. The impact test was carried out according to the PN EN ISO 179.

### 2.10. Mathematical Modeling of Mechanical Properties

The Guth model was used to predict polymer composites’ tensile and flexural modulus. This model is ideal for composites reinforced with non-spherical particles [34,35]. The model equation is:

(5) $$E_{C}=E_{P}\cdot \left(1+0.67\cdot \alpha \cdot V_{f}+1.62\cdot \alpha^{2}\cdot V_{f}{}^{2}\right)$$

where E_C_ is the modulus of reinforced polymer and E_P_ is the modulus for polymer matrix, V_f_ is the volume fraction of the filler, and α is filler particles aspect ratio. The same Equation (5) was used to predict composites’ tensile and flexural modulus. The modulus of the polymer matrix was the tensile and flexural modulus for unreinforced polymer obtained in rotational molding from dry blended powders. The aspect ratio was measured using the optical microscope NIKON Eclipse E 400 (Tokyo, Japan) with camera Meiji Techno HD2600T (San Jose, CA, USA). The measurement was possible because of the computer software connected with the camera.

### 2.11. Thermomechanical Analysis

The dynamic mechanical properties of the samples measuring 10 × 4 × 50 mm^3^ were studied using the DMA method in a torsion mode, operating at a 1 Hz frequency in the temperature range between −125 °C and 110 °C and at a heating rate of 2 °C/min. The measurement were carried out using and Anton Paar MCR 301 rheometer (Graz, Austria). The following equation describes the reinforcement efficiency (r), according to Einstein (6) [36]:

(6) $$r=\frac{\left(\frac{{G’}_{c}}{{G’}_{m}}\right)-1}{V_{f}}$$

where G’_c_ and G’_m_ are storage modulus of composite and unmodified polymeric samples, referred to V_f_, which is the volume fraction of the filler calculated based on the following equation [37]:

(7) $$V_{f}=\frac{\left(\frac{\rho_{m}}{\rho_{f}}\right)}{\left(\frac{1}{M_{f}}\right)+\left(\frac{\rho_{m}}{\rho_{f}}\right)-1}$$

where ρ_m_, ρ_f_ are densities of the matrix and the filler, and M_f_ is the mass fraction of the filler.

## 3. Results and Discussions

### 3.1. Structure Evaluation

**Figure 2:**
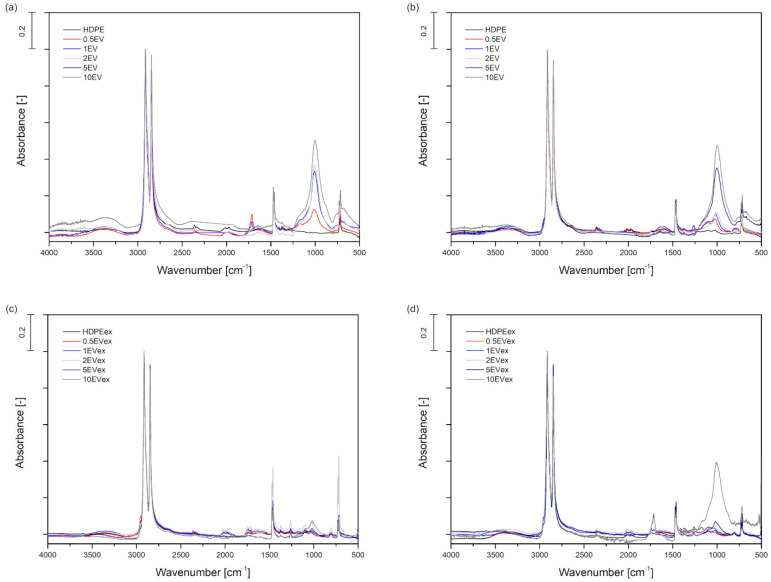
FTIR spectrums of HDPE composites with expanded vermiculite: dry blended measured from outer (**a**) and inner side (**b**); melt compounded measured from outer (**c**) and inner side (**d**).

Thermal and chemical characterization of rotomolded samples was introduced to investigate any changes after incorporating expanded vermiculite. The analysis of the thermal properties from the DSC test showed no significant differences between series containing various EV amounts. The addition of the mineral filler does not change melting temperature and melting enthalpy. All the thermal properties are collected in Table S1 in Supplementary Data. The incorporation of EV to HDPE matrix did not also affect the change of thermal stability of samples. The thermogravimetric test of samples from rotational molding parts showed that all of them are thermally stable up to approximately 435 °C. The curves of TG and DTG are shown in Figure S1 in Supplementary Data. The only dissimilarity between samples is the value of residual mass which is understandable because of the addition of filler. The expanded vermiculite is stable up to 1000 °C, showing only 5% of mass loss [38]. The differences between the mass loss in dry blended samples and melt compounded are related with the insufficient distribution of filler. The mass loss of dry blended samples is much lower than the added content of filler because this method do not provide a good method of compounding the filler into the polymer matrix in the rotational molding process. Table collecting all information from thermogravimetric test is presented in Supplementary Data (Table S2). Temperatures at which the 5%, 10%, and 50% of mass loss occur, the DTG peak values, and the residual mass are included. FTIR was the last test performed to investigate EV’s influence on the properties of rotomolded materials, especially chemical structure. Samples were tested by placing material ones the outer surface and the inner surface of the rotomolded parts toward the beam of infrared radiation. Figure 2 shows the FTIR spectra of samples prepared by dry blending and melt compounding divided due to measurement position. The most significant difference can be seen in the intensity of the peak corresponding to the band Si–O, which is characteristic for vermiculite [39]. This absorption band is approximately 1000 cm^−1^ and is associated with the asymmetric stretching bond between silicon and oxygen in the vermiculite structure [22].

Table 1 presents the values of the intensity for the peak of the Si–O bond. The intensity of this peak is normalized to the intensity of the CH_2_ asymmetric stretching peak at 2914 cm^−1^, which has the biggest intensity [40]. The value of this intensity depends on the surface of the rotomolded samples. According to the data collected in Table 1, the Si–O peak intensity is higher for the inner surfaces of the samples. Additionally, it goes higher with an increasing amount of the expanded vermiculite. It can be stated that the intensity of this peak shows how much of this filler is in samples. The difference between samples only dry blended and melt compounded series is evident. The intensity of peak Si–O is much lower in samples melt compounded, and this peak can be distinguished only for samples containing 2, 5, and 10 wt% EV.

**Figure 3:**
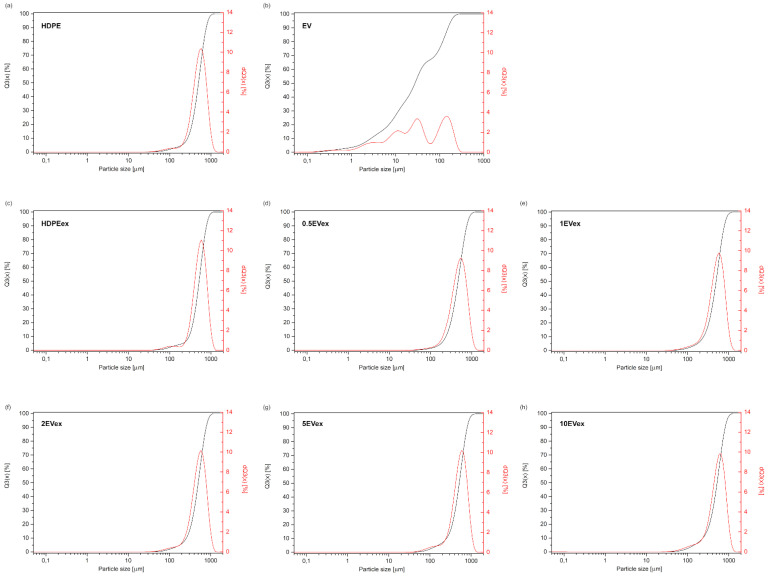
Particle size distribution of polymer powders and expanded vermiculite (EV). (**a**) HDPE, (**b**) EV (expanded vermiculite), (**c**) HDPEex, (**d**) 0.5EVex, (**e**) 1EVex, (**f**) 2EVex, (**g**) 5EVex, (**h**) 10EVex.

In rotational molding technology, the critical aspect is the characterization of polymer powder via the particle size distribution. Figure 3 shows plots of the cumulative size distribution Q3(x) and adequate histogram dQ3(x) of used materials. Polymer powders for rotational molding were obtained in the grinding process using a high-speed mill with a sieve size 500 µm. Figure 3c–h shows particle size distribution graphs for powders obtained through melt compounding, neat extruded HDPE (Figure 3c), and HDPE with expanded vermiculite (Figure 3d–h). The histogram curve is similar for all composite materials powders. The most beneficial particle size distribution is Gaussian like [2], which was achieved for all polymer powders prepared by melt compounding as well as the neat HDPE in the considered case (Figure 3a). The histogram of the particle size distribution for expanded vermiculite (Figure 3b) shows many maxima, which means that this distribution is uneven. This material series has no dominant particle size, but the average size is approximately 27 µm. In this case, this is a trimodal behavior of asymmetric form; the dimensions of particles do not have symmetry between finer and larger particles in relation to the particles of medium size. The same character expanded vermiculite particles distribution is noticed for ball milled and sieved under 74 µm [22].

**Figure 4:**
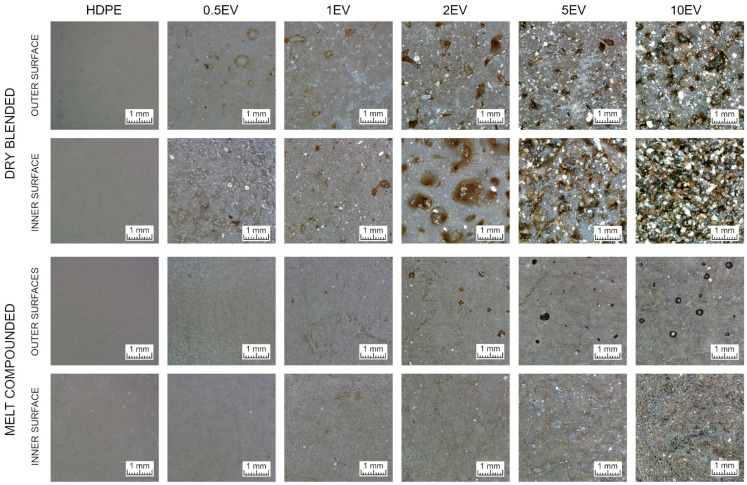
Optical microscope photographs of outer and inner surfaces of rotomolded samples.

Optical microscopy photographs are distinguished into ones taken from the outer side of rotomolded parts and ones from the inner (Figure 4). There is a noticeable difference between those samples’ surface quality and structure. The outer surfaces of dry blended samples are rough with many holes, and with the increasing amount of expanded vermiculite, these surfaces’ unevenness increases. There can be noticed the sparkling gold points, which are related to the external emplacement of EV at the sample surface. In the inner surface of dry blended samples, the holes are more prominent, and there are more gold points of expanded vermiculite. The melt compounded samples have smoother surfaces, although on outer surfaces are holes called pinholes in the rotational molding technology [2]. The gold points of expanded vermiculite can also be seen in melt compounded samples, but they are better embedded in a polymer matrix. This can explain why the Si–O absorption peak intensity originating from EV differs among the sides of samples in FTIR measurement. 

**Figure 5:**
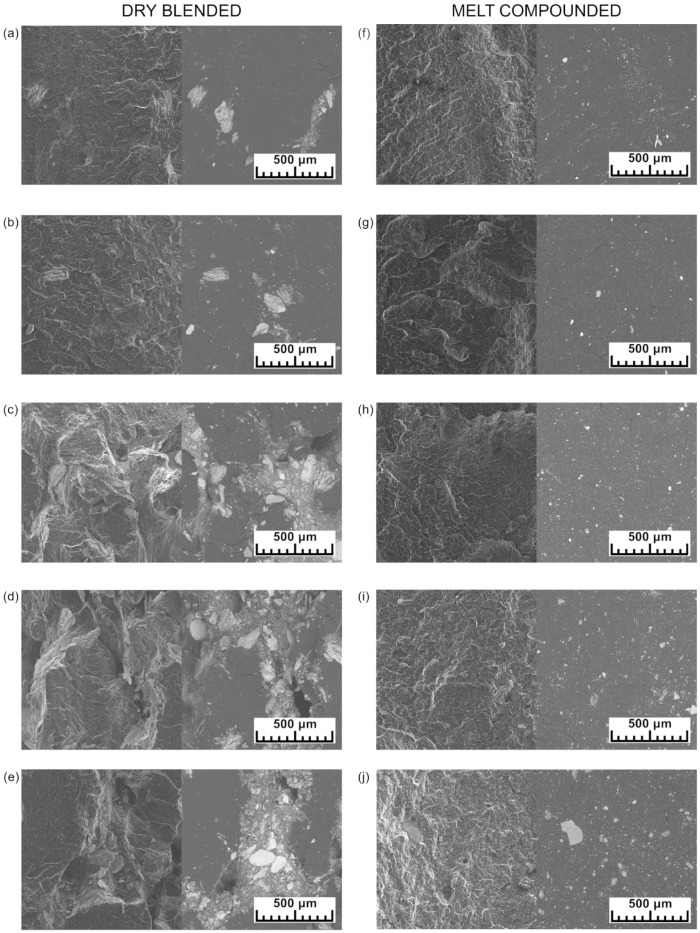
SEM images of cryo-fractured rotomolded samples: dry blended with 0.5 (**a**), 1 (**b**), 2 (**c**), 5 (**d**), and 10 wt% (**e**) of EV and melt compounded with 0.5 (**f**), 1 (**g**), 2 (**h**), 5 (**i**), and 10 wt% (**j**) of EV in the SE and BSE modes.

Figure 5 shows SEM images of cryo-fractured rotomolded samples. Images were taken using SE (left) and BSE (right) detectors. By comparing these two, the surfaces of fractured samples and filler distribution can be seen. Images made using the SE detector show the cross-section of fractured samples. The surface of cryo-fractured dry blended samples is rougher, and the holes are observable; they can be formed by pulling out the filler or by the presence of pores in the structure. The higher the content of the filler, the bigger the holes are. The increasing amount of voids can explain this. The images made using the BSE detector are the best for distribution evaluation because of the high contrast between polymer matrix and filler. The samples made from dry blended powders have large clusters of filler. The expanded vermiculite is combined in large groups, and the big particles of filler are present in the fractured samples. On the other hand, the melt compounded samples have an even filler distribution. There are no big clusters of expanded vermiculite, and the particles are finer. The big particles of expanded vermiculite are seen only in samples with high filler content.

**Figure 6:**
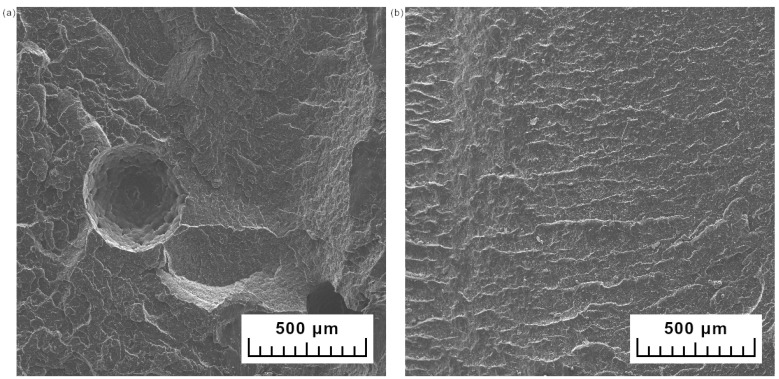
SEM images of cryo-fractured rotomolded samples of neat biobased HDPE made from powder (**a**) and extruded polymer (**b**).

The photographs of cryo-fractured samples of HDPE obtained in two ways are presented in Figure 6. The surface of the cryo-fractured sample of neat ground HDPE is rough and has large pores. In the comparison, the rotomolded series made from extruded HDPE has a smooth fractured surface. The porosity of HDPE samples is almost 3-fold higher than HDPEex (Table 2) and is also proved by the below presented SEM images in Figure 5.

**Figure 7:**
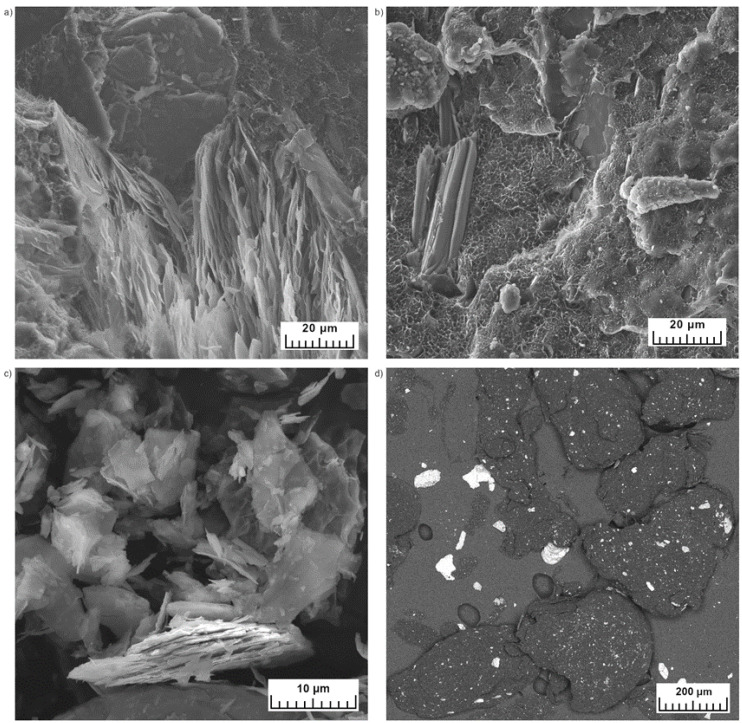
SEM images of cryo-fractured rotomolded composite with 2 wt% dry blended (**a**) and melt compounded (**b**); expanded vermiculite (**c**) and composite powder containing 10 wt% in the BSE mode (**d**).

The brittle fractured rotomolded samples containing 2 wt% are presented in Figure 7a,b. At that magnification, the structure of expanded vermiculite can be observed. Images show the flakes of vermiculite and its concertina shape. The SEM image of EV (Figure 7c) proves that the ground expanded vermiculite has separate plate-like and concertina-shaped particles [23]. This characteristic shape of expanded vermiculite is a structure with an intralayer; vermiculite exfoliates by rapid heating caused by the evaporation of the water from between the sheets [41]. The bigger particles of ground expanded vermiculite still have an intralayer space between plates. The smaller particles reveal the plate shape. In a dry blended rotomolded sample (Figure 6a), the big particles in concertina shape are seen. Separated flakes are primarily present in the fractured surface of the melt compounded sample (Figure 6b); however, there are still the small concertina-shaped particles of EV. In the dry blended composite series, the polymer matrix was not introduced between the layers of expanded vermiculite. In melt compounded samples, the polyethylene matrix covers all of the particles but still some gaps can be caused by the pulling out of the vermiculite. The insufficient interfacial bond between polyethylene matrix and expanded vermiculite is caused by the differences in the polarity between polar inorganic vermiculite and the non-polar polyethylene matrix [29]. This phenomenon is observed for dry blended samples. It is also proved by the mechanical properties which are lowering with the increasing addition of expanded vermiculite in dry blended method. There is a slight increase for samples with the lowest content of expanded vermiculite.

Figure 7d shows the SEM image made in the BSE mode of composite powder with 10 wt% of EV. This picture was taken to investigate how the expanded vermiculite particles will behave after grinding. The filler particles were sieved below 200 µm, and the composite was ground using the 500 µm sieve. The expanded vermiculite is observed inside the polymer powder particles and also outside. The filler is crumbled during grinding from the polymer matrix. From the SEM image made in the BSE mode, it is observed that mainly the bigger filler particles are torn out. Other EDS images are presented in Supplementary Data to prove the same phenomenon (Figure S2). With the increasing amount of EV, there are more pull-out filler particles. The Supplementary Data also presents SE images of composite particles and HDPE particles (Figure S3). The particles have different shapes; there are rounded particles and oblong particles with smoother and rough surfaces but still similar between samples.

An important characteristic of rotomolded products is the porosity resulting from the sintering-based technological process. The presence of micro- and macroscopic voids may strongly affect the mechanical properties of samples because pores act as notches during mechanical load. To calculate the porosity of manufactured parts, the indirect evaluation method based on the samples’ density measurement was used. The theoretical density was calculated using Equation (2). The theoretical density increases with an increasing amount of filler, which is understandable as the density of expanded vermiculite is 2 fold that of neat HDPE. The actual density of powder materials is approximate to the theoretical values. The last measured density is the apparent density of rotomolded samples. This parameter also increases with the addition of the filler for dry blended and melt compounded samples. The only difference between these two samples series is that the melt compounded sample has a higher apparent density, which may directly translate to lower porosity of compounded samples. The melt compounded samples have better mechanical properties and lower porosity, while dry blended samples have higher porosity and worse mechanical properties. This proves the previously mentioned connection between these two characteristics. The porosity among dry blended samples is the highest for samples containing 10 wt% of EV and for melt compounded, but it is approximately 2-fold lower.

**Table 2:** The densities of materials used to prepare rotomolded samples and porosity in rotomolded samples.

Sample		Theoretical Density ^1^	Actual Density ^2^	Apparent Density ^3^	Porosity
		[g/cm^3^]			[%]
Dry blended	HDPE	0.9563	-	0.9135 ± 0.0074	4.48 ± 0.78
	0.5EV	0.9592	-	0.9369 ± 0.0006	2.32 ± 0.06
	1EV	0.9620	-	0.9380 ± 0.0009	2.50 ± 0.10
	2EV	0.9679	-	0.9224 ± 0.0015	4.70 ± 0.15
	5EV	0.9861	-	0.9266 ± 0.0023	6.03 ± 0.24
	10EV	1.0191	-	0.9528 ± 0.0147	6.50 ± 1,44
Melt compounded	HDPEex	-	0.9564 ± 0.0842	0.9400 ± 0.0019	1.71 ± 0.20
	0.5EVex	-	0.9642 ± 0.0618	0.9387 ± 0.0020	2.64 ± 0.21
	1EVex	-	0.9646 ± 0.0987	0.9426 ± 0.0031	2.28 ±0.32
	2EVex	-	0.9735 ± 0.0702	0.9422 ± 0.0026	3.22 ± 0.27
	5EVex	-	0.9895 ± 0.0794	0.9566 ± 0.0021	3.32 ± 0.21
	10EVex	-	1.0254 ± 0.0675	0.9917 ± 0.0039	3.29 ± 0.38
Expanded vermiculite		-	2.3341 ± 0.0893	-	-

^1^ Density calculated using Equation (2); ^2^ density obtained from measurement using gas pycnometer; ^3^ density calculated using Equation (5).

### 3.2. Processing Parameters

Among the most critical processing properties of polymer materials considered in selecting materials for rotational molding is the melt flow index. The preferred MFI should be between 2 and 8 g/10 min [7]. The MFI declared by the producer of HDPE is 7.2 g/10 min; however, the measured MFI is slightly higher than the upper bound of the range and decreases with the addition of the filler (Table 3). Usually, the addition of reinforcement into the polymer matrix increases melt viscosity, which indirectly lowers the melt flow index [42]. The observed changes in the MFI are negligible; therefore, it can be stated that the addition of up to 10 wt% of the EV, should not affect the processing properties of these composites through the rotational molding technology. All samples were prepared successfully according to the visual appearance of the samples (Figure 1). However, the increased porosity may be related to increased viscosity at low shear rates, which decreases the ability to air entrapment removal during the melting process [43].

### 3.3. Mechanical and Thermomechanical Properties

The evaluation of the tensile and flexural properties shows changes between the mechanical properties for samples obtained by only dry blending the polymer matrix with filler and the rotomolded composites from previously melt compounded materials. The Charpy impact test was also carried out to compare the mechanical properties of rotomolded samples further. The toughness from the tensile test was calculated as the area under the stress–strain curve.

**Figure 8:**
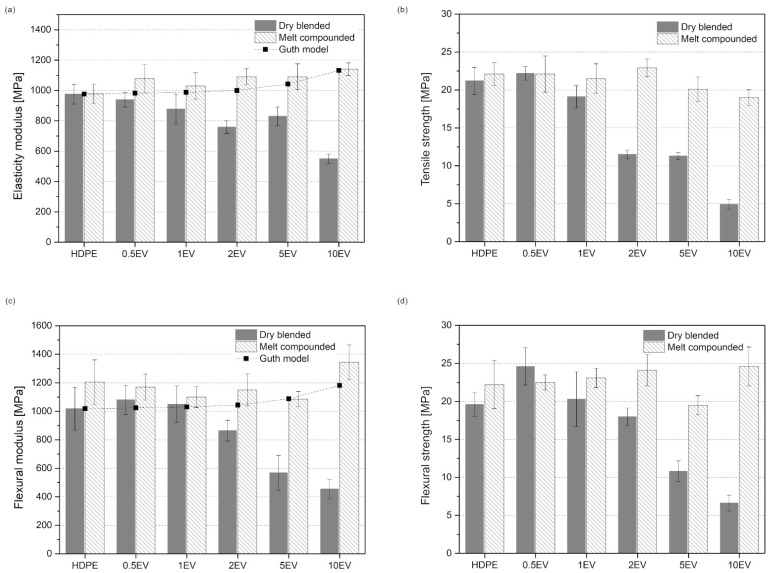
Mechanical properties of composite samples from the tensile test: elasticity modulus (**a**) and tensile strength (**b**), and from flexural bending: flexural modulus (**c**) and flexural strength (**d**).

Figure 8 presents the tensile and flexural properties of obtained composites plotted with the mathematical model values according to Equation (5). The tensile strength decreases with the increasing amount of filler. For samples obtained by dry blending polymer matrix with a filler, the difference between a neat sample and 10EV is approximately 16 MPa. The tensile strength value for compounded composite powders decreases with increasing filler content, but only by 3 MPa. The greater the filler share, the more significant differences in tensile strength value. It should be noticed that the 10EVex reveals more than 400% higher tensile strength than 10EV. The elasticity modulus for dry blended series decreases with the increasing amount of expanded vermiculite. Different behavior is observed for samples compounded; the elasticity modulus slightly rises with the increase in the amount of filler. The elasticity modulus for the sample with 10 wt% EV is approximately 2-fold higher for rotomolded samples obtained from melt compounded polymer composite powder. The increase in the elastic modulus connected with the decrease in tensile strength is typical for thermoplastic polymers filled with inorganic powder fillers [44]. This behavior can be observed for samples produced in the two-step method, but for samples obtained from dry blended powders, the values of elastic modulus and tensile strength decline.

In a three-point bending test, the flexural modulus and strength were evaluated. The flexural strength for samples obtained only by dry blending decreases with the increasing filler content. This value slightly goes higher for melt compounded samples; the only exception is samples 5EVex. The highest flexural strength is 24.6 MPa for 10EVex, and the lowest is 19.5 MPa for 5EVex. The flexural modulus for melt compounded samples is relatively steady, the only increase is for sample 10%EVex, and the value for flexural modulus is 1344 MPa. The value for flexural modulus for melt compounded samples decreases with the increasing amount of filler. The flexural properties are better for melt compounded samples. The most significant difference is between sample series with 10 wt% of EV; for flexural modulus, it is 889 MPa, and for flexural strength, almost 18 MPa.

The mathematical modeling using the Guth model helps predict values of the tensile and flexural modulus of composites. The calculated values from Equation (1) are plotted with the elasticity and flexural modulus (Figure 3). The comparison between theoretical and measured values shows that tensile and flexural modulus for melt compounded samples are more comparable with the Guth model values. To better investigate the similarity and dissimilarity between theoretical and measured values, Pearson’s correlation coefficient (r) was calculated. The correlation coefficient for tensile and flexural modulus values for dry blended samples are −0.91 and −0.92, respectively. The same values for melt compounded samples are 0.77 for tensile modulus and 0.675 for flexural modulus. This means that the moduli values obtained in the experiment for samples prepared in the two-step process are more closely related to the theoretical values. This is called positive correlation. It means that when theoretical values increases, the experimental also increases. The negative value of the correlation coefficient means the opposite. The better correlation for melt compounded samples is reasonable when the lowered amount of structural defects is taken into account.

**Figure 9:**
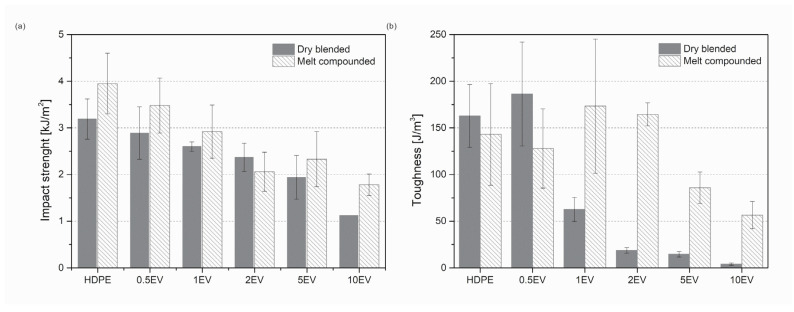
The impact strength for samples from the Charpy impact (**a**) test and measured toughness (**b**).

The impact strength for notched samples does not show such significant differences between dry blended and melt compounded samples (Figure 9a). Impact strength for all samples decreases with the increasing amount of expanded vermiculite. The dry blended series shows slightly lower impact strength except for sample 2EV. Another way to measure the material’s strength is to calculate the toughness from the tensile test [33]. The calculated toughness values are expressed in the column graph in Figure 9b. This value drastically decreases for dry blended samples when the amount of filler increases to 1 wt% EV. This means that the energy needed to break the specimen in an uniaxial tensile test decreases. For melt compounded parts, the toughness goes slightly higher for 1EV and 2EV; then, this value decreases with the increasing amount of filler. Differences among samples dry blended and melt compounded are seen in neat samples and containing 0.5% EV. Their toughness is a bit higher for dry blended samples, but with the growth of the filler amount, the toughness for melt compounded samples increases. These two methods of determining the toughness show the same tendency: the decrease in toughness value with the increasing amount of the filler. All performed mechanical test proves the hypothesis that dry blended rotomolded composites reveal significantly lower mechanical strength.

**Figure 10:**
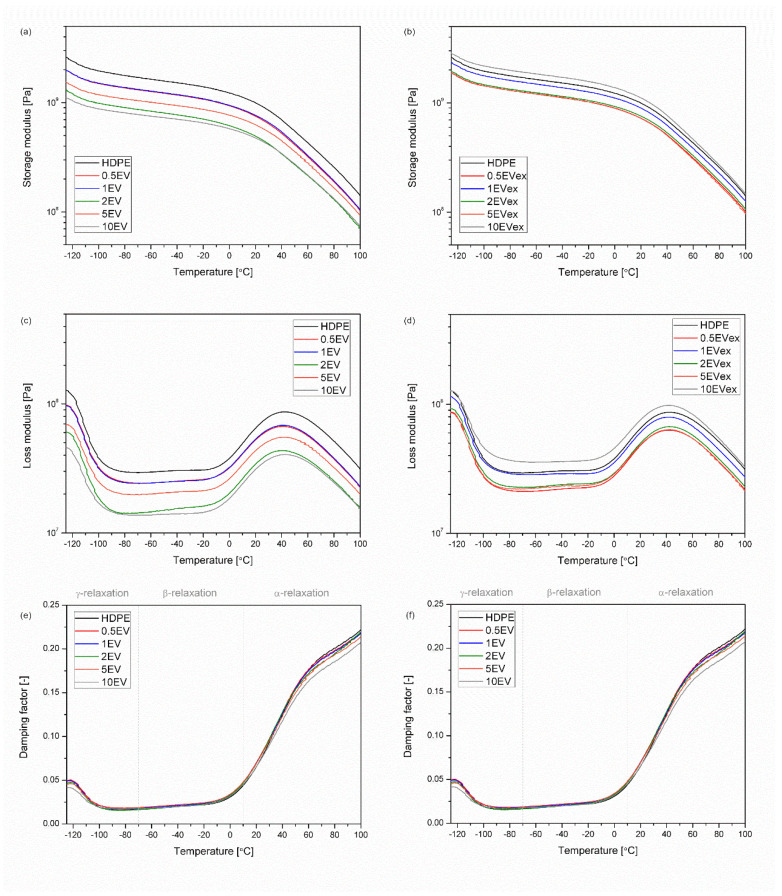
DMA results, storage (G’) (**a**,**b**), loss (G”) (**c**,**d**) moduli, and damping factor (tanδ) (**e**,**f**) vs. temperature.

Figure 10 shows the changes in storage modulus (G’) and damping factor (tanδ) as a function of temperature obtained by the DMA. Additionally, information about the peak of G” curves, glass transition temperature determined as a local maximum of tanδ measured in the range of γ-relaxation, and reinforcing efficiency calculated according to Equation (6) are collectively presented in Table 4. The correlation between changes in the elasticity modulus determined in the static tensile test and changes in the storage modulus assessed by DMA is known and described in the literature [36]. In the considered case, for both material series, i.e., manufactured by single-step processing with physically mixed blends and with micro pellets, a trend in the change of G’ values is observed, similar to the results obtained in the tensile test. However, it should be noted that when assessing thermomechanical parameters determined in dynamic conditions, the curves contain more complex information about the properties and interactions of the components that make up the composite. The introduction of EV in the form of powders to the polymer matrix by dry blending resulted in numerous agglomerates and significant porosity, which translated into a deterioration of the rotationally molded part stiffness. For material series made of melt compounded materials, G’ gradually decreased with the increasing filler share in the composite. Still, the highest rigidity was noted for the sample containing the highest EV content (10EVex). The G” curves reveal the typical course for high-density polyethylene. The G”(T) curve shows the peak of γ-relaxation resulting from the reorganization of loose chain ends (defects) in the polymer crystals in the range below −100 °C, almost invisible β-relaxation in the range between −35 and −5 °C (related to amorphous phase with motion of the branches), and α-relaxation, above 20 °C, which is generally representative of the crystalline phase and originates from some motion in the crystals [45]. In the dry blended series, it is impossible to observe the trends in changes in the shifts of the relaxation peaks, the most detailed and the most visible α-relaxation. However, for all composites, melt blended samples showed a slight shift of the G”(T) curve peak in α-relaxation. Considering the lack of changes in the degree of crystallinity induced by the presence of the filler, this trend can be attributed to the presence of rigid filler particles, with proper adhesion resulting from interactions between the polymeric matrix and filler particles [46]. Analysis of tanδ(T) curves indirectly allows the assessment of the damping properties of materials. In the case under consideration, the introduction of fillers did not cause almost any changes in the course of the curves. The only noticeable differences were noted in terms of the softening point of the material above the α-relaxation for a series of products made with the dry blending method. For composites with the highest filler concentrations, a slight decrease in the tanδ value in the range of the highest temperature values was noted. While these composites were characterized by the highest porosity, which should increase the ability for mechanical attenuation of the material [47], the presence of agglomerated rigid structures of the inorganic filler compensated and dominated this effect, leading to a reduction tanδ values, which is synonymous with a reduced ability to damp mechanical vibrations in the current temperature range. Taking into consideration determined values of the porosity, and increased share of inorganic particles with low aspect ratio, as can be expected, reinforcing efficiency r was lowered with increasing filler content. What should be noticed is the fact that r values do not vary between three different temperatures, which may be understood by the lack of the influence on the macromolecular structure of the polyethylene, and as it was mentioned in case of thermal properties discussion, negligible nucleating ability of EV on HDPE. The higher r values determined for dry blended series, in this case, do not result from the increased efficiency and action of the filler but from the reduced proportion of the polymer and the agglomerated filler structures in the composite matrix.

## 4. Conclusions

As part of this work, a series of composite products based on biobased polyethylene filled with ground expanded vermiculite were successfully produced using rotational molding technology. This study aimed to obtain information on the legitimacy of using preliminary melt processing of the composites and compare the composites produced in this way with the series shaped by the one-step powder mixing process. The investigation results suggest that the melt compounding method allows obtaining rotomolded parts characterized by good mechanical properties. These results in reduced structural defects and good filler dispersion, making this method justified despite the increased number of technological processes and consumed energy. All evaluated mechanical properties, including tensile, flexural, and impact strength, are higher for melt compounded samples.

Moreover, the quality of melt compounded rotomolded samples is better than that of dry blended samples. The higher porosity of dry blended samples also causes lower quality and strength of rotomolded parts. Dry blended samples revealed a lot of visible filler particles, especially from the inner surface. The spectroscopic analysis allows correlation of the observed changes in the intensity of Si–O peak originated absorption bands with the dispersion level of the filler. FTIR evaluation shows that agglomerated filler structures observed at the part surface are higher for dry blended samples from the inner surface. Expanded vermiculite is better distributed in melt compounded samples, and the intensity of the Si-O peak is lower for these samples. From the point of view of processing properties, incorporating EV into the HDPE matrix did not significantly change the melt flow index of composites compared to the unmodified polymer. Therefore, EV seems to be a perspective filler to manufacture the rotationally molded thin-walled composite parts, which does not cause deterioration of processability of materials. In conclusion, the composite rotomolded parts have better quality when prepared by melt compounding the filler with polymer matrix.

## Figures and Tables

**Table 1:** The intensity of the FTIR absorption peak related to the Si–O bond.

Sample		Intensity (-)	
		Outer Surface	Inner Surface
Dry blended	HDPE	-	-
	0.5EV	0.0738	0.1264
	1EV	0.1009	0.3328
	2EV	0.1119	0.3693
	5EV	0.3524	0.5022
	10EV	0.4740	0.9382
Melt compounded	HDPEex	-	-
	0.5EVex	-	-
	1EVex	-	-
	2EVex	-	0.0419
	5EVex	0.0330	0.0709
	10EVex	0.0745	0.3931

**Table 3:** The melt flow index for composite samples obtained in melt compounding.

Sample		MFI
		[g/10 min]
Melt compounded	HDPEex	9.30 ± 0.37
	0.5EVex	9.28 ± 0.35
	1EVex	9.10 ± 0.71
	2EVex	9.32 ± 0.59
	5EVex	8.46 ± 0.36
	10EVex	8.30 ± 0.31

**Table 4:** Thermomechanical parameters obtained from DMA data.

Sample		Tg G”α [°C]	tanδ at Tg (-); [°C]	r (-)		
				−80 °C	25 °C	80 °C
Dry blended	HDPE	42.2	0.0578; −130	-	-	-
	0.5EV	41.5	0.0503; −122	1.47	1.58	1.73
	1EV	41.0	0.0493; −122	0.68	0.72	0.79
	2EV	40.2	0.0475; −122	1.16	1.23	1.15
	5EV	42.3	0.0461; −122	0.30	0.28	0.26
	10EV	42.6	0.0416; −123	0.27	0.24	0.21
Melt compounded	HDPEex	39.8	0.0478; −123	-	-	-
	0.5EVex	41.8	0.0469; −123	2.26	2.05	1.64
	1EVex	41.9	0.0498; −123	0.47	0.36	0.21
	2EVex	42.2	0.0484; −122	0.52	0.43	0.32
	5EVex	42.9	0.0467; −122	0.23	0.20	0.17
	10EVex	41.8	0.0455; −122	−0.01	−0.02	−0.02

## Data Availability

The data presented in this study are available on request from the corresponding author.

## Supplementary Materials

The following supporting information can be downloaded at: https://www.mdpi.com/article/10.3390/ma15175903/s1, Table S1. Thermal properties from DSC of samples obtained in rotational molding technology; Table S2. Thermogravimetric properties; Figure S1. TG (a) and DTG (b) curves obtained from thermogravimetric measurement; Figure S2. EDS SEM images of composite powder particles containing 0.5 (a), 1, 2 and 5 wt% of EV; Figure S3. SEM images of HDPE (a) and HDPEex (b) and composite particles containing 0.5 (c), 1 (d), 2 (e), 5 (f), and 10 wt% (g).
