# Supplementary material for: The Relationship between a Rotational Molding Processing Procedure and the Structure and Properties of Biobased Polyethylene Composites Filled with Expanded Vermiculite
Source: Materials (Basel). 2022 Aug 26;15(17):5903. doi: 10.3390/ma15175903 (PMC9457396; doi:10.3390/ma15175903)
Supplement: Supplementary file 1 [file materials-15-05903-s001.zip › materials-1826837-supplementary.pdf]

materials

Article

The Relationship between a Rotational Molding Processing Procedure and the Structure and Properties of Biobased Polyethylene composites Filled with Expanded Vermiculite

Joanna Aniśko [1,*], Mateusz Barczewski [1], Adam Piasecki [2], Katarzyna Skórczewska [3], Joanna Szulc [3] and Marek Szostak [1]

[1] Institute of Materials Technology, Faculty of Mechanical Engineering, Poznan University of Technology, Piotrowo 3, 61-139 Poznan, Poland
[2] Institute of Materials Engineering, Faculty of Materials Engineering and Technical Physics, Poznan University of Technology, Piotrowo 3, 61-138 Poznan, Poland
[3] Faculty of Chemical Technology and Engineering, Bydgoszcz University of Technology, Seminaryjna 3, Bydgoszcz, Poland
* Correspondence: joanna.anisko@put.poznan.pl

1. Supplementary data

Table S1. Thermal properties from DSC of samples obtained in rotational molding technology.

		T_M	T_C	ΔH_M	X_C (1)
		[°C]		[J/g]	[%]
**Dry blended**	**HDPE**	132,2	117,2	195,6	66,76
	**0,5EV**	133,2	116,2	188	64,49
	**1EV**	133,4	116	178,1	61,40
	**2EV**	134,6	116,4	184,9	64,39
	**5EV**	133,7	114,7	178,5	64,13
	**10EV**	134,1	114	180,4	68,41
**Compounded**	**HDPE**	133,3	117,2	189,9	64,81
	**0,5EV**	132,9	117,9	196	67,23
	**1EV**	133,6	117,7	184,7	63,67
	**2EV**	133,7	118	191	66,52
	**5EV**	133	118,3	185	66,46
	**10EV**	132,8	118,6	177,3	67,24

$$X_C = \frac{\Delta H_M}{(1-\varphi)\cdot\Delta H_{100\%}} \cdot 100\% \quad (1)$$

ΔH_M – melting enthalpy [J/g]
$\Delta H_{100\%}$ - melting enthalpy of 100% crystalline PE (293 J/g)
φ – content of the filler [-]

Table S2. Thermogravimetric properties.

		**W_5**	**W_{10}**	**W_{50}**	**DTG**		**ΔW**
			[°C]		[°C]	%/min	[%]
Dry blended	HDPE	433	444,7	468,9	470,9	38,1	-1,37
	0,5EV	433,7	445,8	469,3	474,5	35,03	-0,04
	1EV	433,1	445	469,1	473,6	35,2	0,41
	2EV	434,5	446,5	469,3	472,9	37,08	2,71
	5EV	435,3	446,7	470,2	474,4	32,75	4,83
	10EV	433,4	445,5	469,8	472,6	30,85	5,12
Compounded	HDPE	437,5	449	471,9	476,1	37,63	-0,6
	0,5EV	436,3	447,8	471,4	476,8	35,52	0,44
	1EV	435	447,2	471,3	476,6	35,02	0,36
	2EV	436,4	448,8	473	478,2	34,94	1,5
	5EV	435,3	448,1	472,1	472,4	33,4	4,47
	10EV	435,2	447,3	472,4	475	32,18	8,99

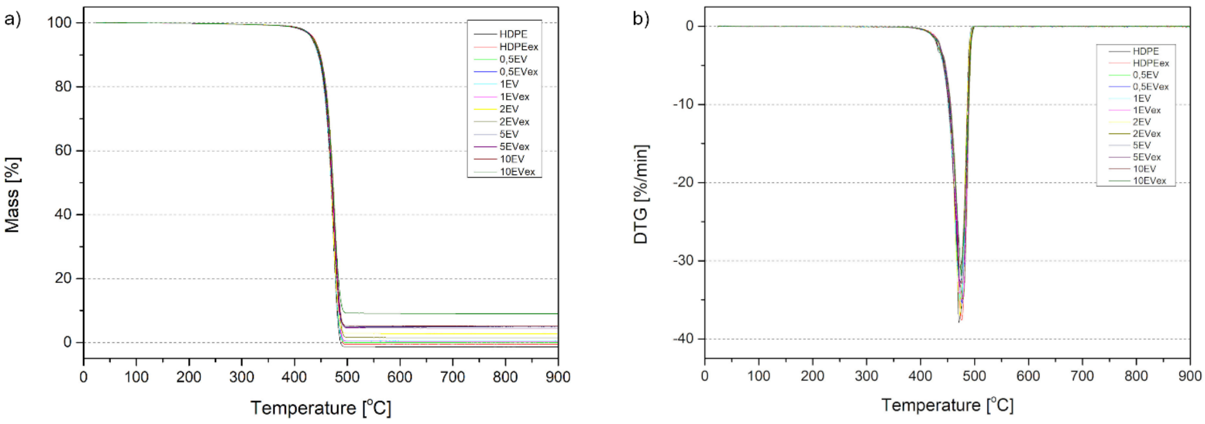

Figure S1. TG (a) and DTG (b) curves obtained from thermogravimetric measurement.

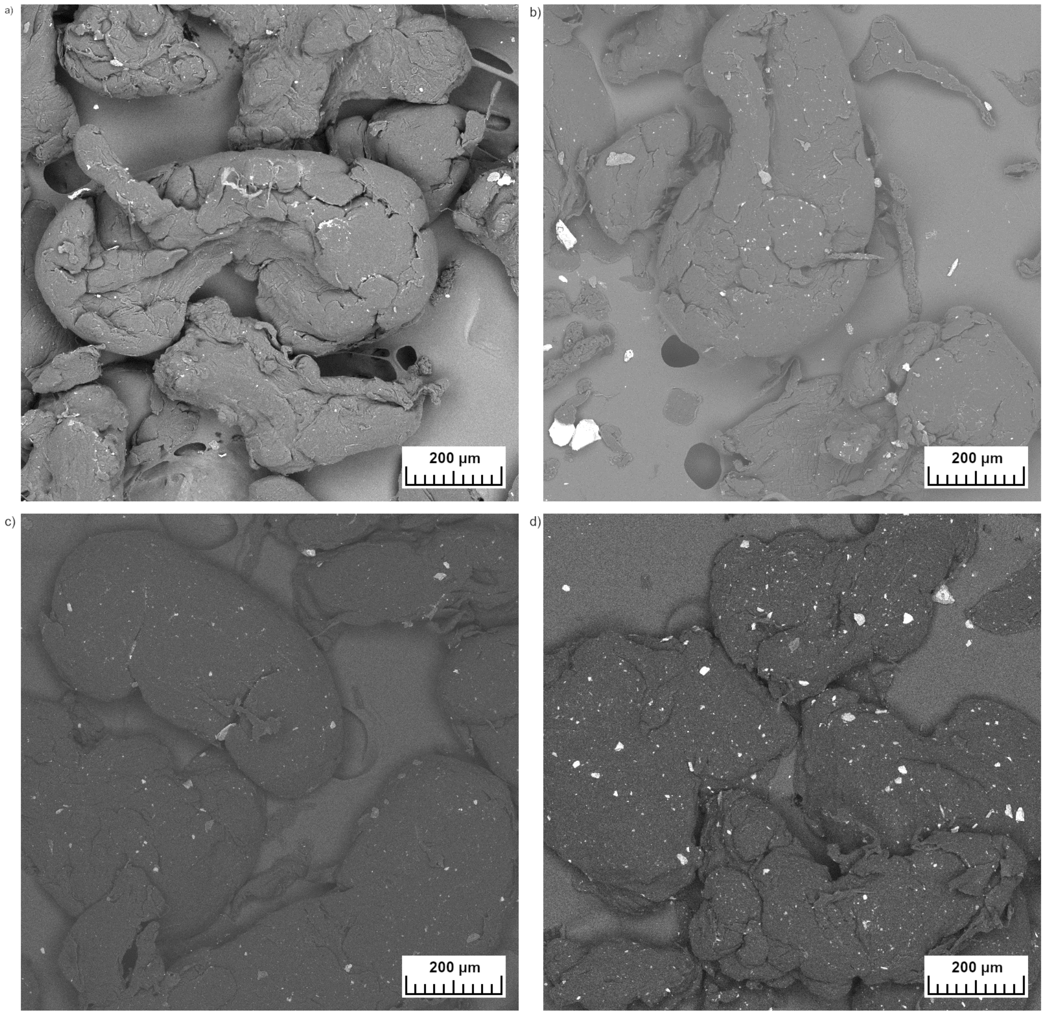

Figure S2. EDS SEM images of composite powder particles containing 0.5 wt% (a), 1 wt%, 2 wt% and 5 wt% of EV.

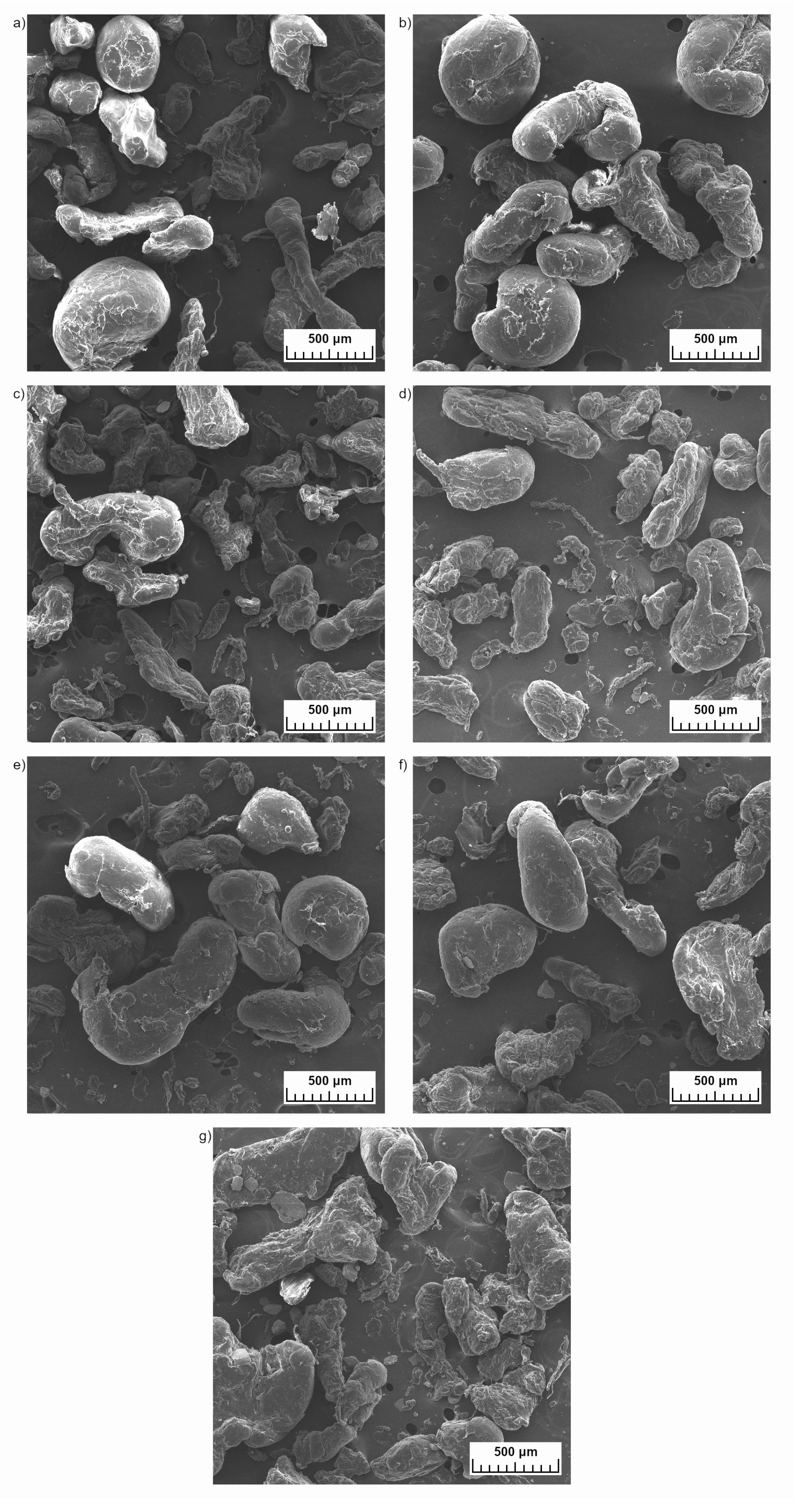

Figure S3. SEM images of HDPE (a) and HDPEex (b) and composite particles containing 0.5 wt% (c), 1 wt% (d), 2 wt% (e), 5 wt% (f), 10 wt% (g).
